# Advancements of CRISPR-Mediated Base Editing in Crops and Potential Applications in *Populus*

**DOI:** 10.3390/ijms25158314

**Published:** 2024-07-30

**Authors:** Xuefei Yang, Ping Zhu, Jinshan Gui

**Affiliations:** State Key Laboratory of Subtropical Silviculture, Zhejiang A&F University, Hangzhou 311300, China; xfyang@stu.zafu.edu.cn (X.Y.); pzhu@stu.zafu.edu.cn (P.Z.)

**Keywords:** CRISPR-Cas9, genome editing, base editor, crop, *Populus*

## Abstract

Base editing represents a cutting-edge genome editing technique that utilizes the CRISPR system to guide base deaminases with high precision to specific genomic sites, facilitating the targeted alteration of individual nucleotides. Unlike traditional gene editing approaches, base editing does not require DNA double-strand breaks or donor templates. It functions independently of the cellular DNA repair machinery, offering significant advantages in terms of both efficiency and accuracy. In this review, we summarize the core design principles of various DNA base editors, their distinctive editing characteristics, and tactics to refine their efficacy. We also summarize their applications in crop genetic improvement and explore their potential contributions to forest genetic engineering.

## 1. Introduction

Genome editing is a powerful genetic engineering tool that can precisely modify target genome sites [1]. This technology employs single guide RNA (sgRNA) to direct engineered nucleases to specific loci, resulting in site-specific double-strand breaks (DSBs). These breaks prompt organisms to introduce insertions or deletions at the target site through repair mechanisms, such as non-homologous end joining (NHEJ) or homologous recombination repair (HDR). However, HDR is often inefficient, and NHEJ typically produces frameshift mutations, leading to protein inactivation or loss of function. Consequently, traditional genome editing methods struggle to achieve precise single-base replacements.

Superior allelic variations in important agronomic traits in crops are frequently due to single-base variations rather than complete gene function loss. For instance, novel Wx alleles generated by base editing have improved rice grain quality [2], and base-editing-mediated artificial evolution of OsALS1 has developed novel herbicide-tolerant rice germplasms [3]. Additionally, the lengthy reproductive cycles of forest trees limit the speed and effectiveness of traditional genetic enhancement. Therefore, the development of a precise and efficient single-base editing system is crucial for genetic improvement in forestry.

Base editing technology is an innovative gene editing method that utilizes CRISPR-Cas to precisely target specific DNA sequences and achieve accurate base replacement through the chemical modification of single nucleotides catalyzed by deaminase. This technology eliminates the need for double-strand breaks (DSBs) or reliance on endogenous DNA repair mechanisms, offering efficient, precise, and widely applicable advantages. This review provides a comprehensive overview of the developmental history, operational principles, research advancements, and potential applications of DNA base editing technology in forest genetic improvement.

## 2. Background and Mechanisms of Base Editing Technology

The CRISPR-Cas system is an adaptive immune mechanism found in prokaryotes, providing defense against viral invasions [4]. Discovered and named by Jansen et al. in 2002, Clustered Regularly Interspaced Short Palindromic Repeats (CRISPR) sequences, along with Cas proteins, form the CRISPR-Cas system [5]. This system integrates fragments of foreign viral DNA into the spacer regions of the CRISPR sequence [6]. Upon subsequent virus invasion, the system recognizes and cleaves the viral DNA, thereby inactivating the virus and achieving immunity [7].

CRISPR-Cas9 gene editing technology primarily employs the Cas9 protein in combination with sgRNA to recognize and cut specific DNA sequences, inducing the organism to introduce insertion or deletion mutations at the target site through non-homologous end joining (NHEJ) or homologous recombination repair (HDR) [8]. Although HDR can achieve more precise insertion of the target sequence compared to NHEJ, its efficiency is low, and it is limited by the cell cycle phase [9]. The potential for DSBs to induce off-target mutations, leading to protein inactivation or loss of function, limits the precision of traditional gene editing [10]. However, controlling important agronomic trait genes typically involves single-base mutations.

Base editing technology was developed to address this concern. This technology uses CRISPR-Cas to target specific DNA sequences and achieves precise base replacement through deaminase-catalyzed chemical modification of single nucleotides. It operates without the need for DSBs and does not rely on endogenous DNA repair mechanisms, offering efficient, accurate, and widely applicable advantages.

**Figure 1:**
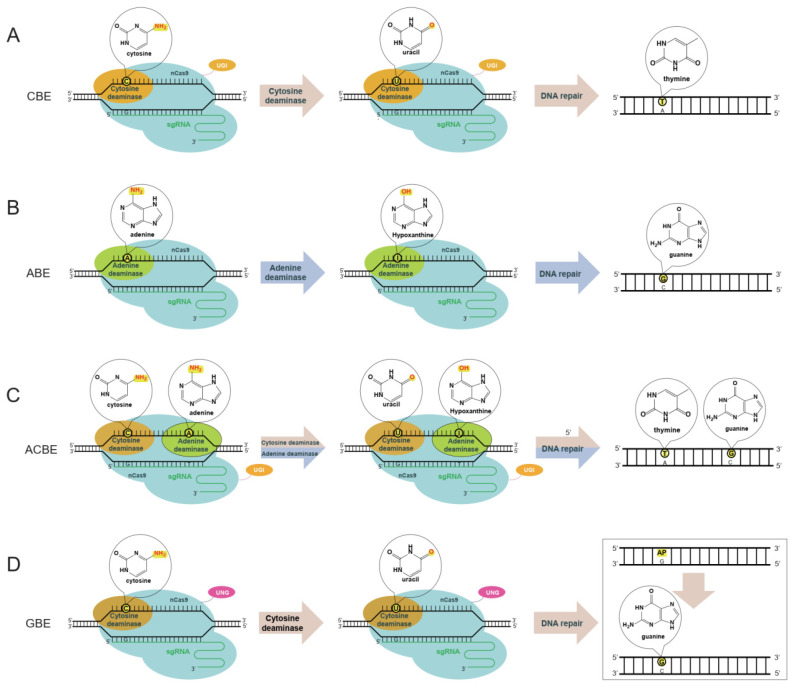
Types of base editors and their principles of action. (**A**) Cytosine base editor (CBE) uses cytosine deaminase to convert cytosine (C) to uracil (U), thereby achieving C to T editing; (**B**) Adenine base editor (ABE) uses adenine deaminase to convert adenine (A) to inosine (I), thereby achieving A to G editing; (**C**) Dual base editor (ACBE) combines CBE and ABE’s functions, allowing for both C to T and A to G editing; (**D**) Glycosylase Base Editor (GBE) uses cytosine deaminase and uracil glycosylase (UNG) to convert C to U, and then de-glycosylates U to a uracil nucleotide (dU) to allow for C to G editing.

Since the development of the first base editor (BE1) by Komor et al. in 2016, a series of DNA base editors have been rapidly developed (Figure 1) [11,12]. For example, the cytidine base editor (CBE) can convert cytosine (C) and guanine (G) base pairs to thymine (T) and adenine (A) base pairs (Figure 1A) [11]; The adenine base editor (ABE) can convert adenine (A) and thymine (T) base pairs to guanine (G) and cytosine (C) base pairs (Figure 1B) [13]; The dual base editor (DBE) can simultaneously achieve conversion of both C–G base pairs and A–T base pairs (Figure 1C) [14]. These editors are fusion proteins composed of Cas protein, deaminase, and other auxiliary factors. Guided by sgRNA to recognize specific PAM sequences, they direct the deaminase to the target site. The deaminase enzyme catalyzes the deamination of cytosine (C) or adenine (A), resulting in the formation of uracil (U) or inosine (I). During DNA replication, these modified bases are recognized as thymine (T) or guanine (G), respectively, effectively accomplishing base conversion.

Recently, a new type of DNA base editor called the Glycosylase base editor (GBE) has been discovered. It can convert C–G base pairs to G–C base pairs (Figure 1B) [15,16]. The GBE utilizes a glycosylase enzyme (single-strand selective uracil DNA glycosylase), which removes U from DNA, creating an abasic site (AP site) lacking a base. The AP site is then repaired as G, resulting in a C to G base conversion. GBE is also a fusion protein composed of Cas protein, uracil DNA glycosylase (UNG), and other auxiliary factors. Initially, it employs a cytidine deaminase enzyme to convert cytosine (C) to uracil (U). Subsequently, the uracil is eliminated, creating an AP site within the genome. Finally, cellular repair mechanisms repair the AP site, resulting in the substitution of guanine (G). Unlike CBE and ABE, which typically mediate conversions among chemically similar bases (e.g., purine to purine, pyrimidine to pyrimidine), GBE offers the capability to achieve base conversions between purines and pyrimidines, significantly enhancing the versatility and utility of genome base editing techniques.

## 3. Types and Optimization of DNA Base Editors

### 3.1. Cytidine Base Editor (CBE)

In 2016, Komor et al. developed the Cytidine Base Editor (CBE), which enables the conversion of C–G base pairs to T–A without generating double-strand breaks (DSBs) (Table 1) [11,17]. They integrated the cytidine deaminase APOBEC1, which is derived from rats, with the dCas9 protein, which is a variant of the Cas9 protein containing point mutations Cas9D10A and H840A. These mutations preserve the DNA binding functionality while abolishing the DNA cleavage capability, leading to the creation of the initial cytidine base editor known as Base Editor 1 (BE1) [11]. BE1 employs sgRNA to direct the dCas9 protein to the target DNA sequence, facilitating the separation of the double-stranded DNA to create a single-stranded R-loop structure. Within this R-loop, the cytosine residue serves as a substrate for the cytidine deaminase domain, undergoing enzymatic deamination to convert it to uracil. Subsequently, DNA repair processes insert an adenine base on the complementary strand opposite the uracil, completing the modification. Uracil DNA glycosylase then removes the uracil, and thymine is inserted into the deaminated strand, successfully converting C–G to T–A. However, the BE1 system exhibits relatively limited editing efficiency due to the uracil DNA glycosylase’s capacity to excise the uracil from the deaminated strand before the guanine on the non-deaminated strand is transformed into adenine. This premature removal of uracil results in the formation of an abasic site, which triggers DNA repair mechanisms that restore the original sequence.

To improve editing efficiency, Komor et al. attached a uracil DNA glycosylase inhibitor (UGI) to the C-terminus of dCas9 in BE1, creating the second-generation cytidine base editor, BE2 [11]. Subsequently, the team substituted the dCas9 protein in BE2 with nCas9, a mutated form of Cas9 protein carrying the Cas9D10A mutation. This mutation retains the DNA binding functionality of Cas9 while enabling the HNH nuclease domain to introduce a single-strand nick on the complementary strand. This modification further enhanced base editing efficiency, leading to the development of the third-generation cytidine base editor, BE3. To further optimize the system, Komor et al. appended an additional UGI adjacent to the first UGI and incorporated nuclear localization signals at both termini of BE4, achieving even greater editing efficiency [18,19].

In 2017, Zong et al. optimized the codons in BE3, resulting in the base editor nCas9-PBE [20]. Due to the inherent preference of APOBEC1 for specific cytosines, which limits the editing range and efficiency of CBE, replacing APOBEC1 with the cytidine deaminase PmCDA1 from lampreys effectively expanded the editing range and improved the editing efficiency of CBE [21]. In 2023, Neugebauer and his team announced the latest advancements in CBE systems. They employed phage-assisted continuous evolution techniques to engineer a deoxyadenosine deaminase, TadA-8e, which exhibits exceptional activity. This enzyme was successfully fused with dCas9 to develop TadCBEs [30]. TadCBEs employ the enzyme deoxyadenosine deaminase to catalyze the deamination of cytidine, generating a CBE with reduced dimensions and diminished off-target effects, thereby enhancing both precision and editing efficiency. Subsequently, Yang et al. capitalized on the deaminase hAPOBEC3A to establish the A3A-BE4max system. Through mutagenesis of hAPOBEC3A, they devised numerous iterations of this system. Following rigorous screening, they identified a mutant variant with the lowest off-target efficiency, ultimately yielding the haA3A-CBE [31].

### 3.2. Adenine Base Editor (ABE)

In 2017, Gaudelli, Komor, and colleagues reported the development of a new adenine base editor (ABE), which enables the conversion of A–T base pairs to G–C without generating DSB (Table 1) [13]. ABE employs the *Escherichia coli* tRNA adenosine deaminase enzyme, designated as ecTadA. By integrating ecTadA with the nCas9 protein and the TadA dimer, and through seven iterations of refinement, they successfully developed the adenine base editor ABE7.10.

The mechanism of ABE7.10 operates as follows: sgRNA guides the nCas9 protein to the target gene site, where it binds and cleaves the double-stranded DNA, creating a single-stranded R-loop structure. Within this R-loop, adenine residues undergo deamination catalyzed by TadA, converting them into inosine. During subsequent DNA replication, inosine is interpreted as guanine, and the DNA repair machinery incorporates cytosine into the complementary strand. Consequently, the original A–T base pair is replaced with a G–C base pair [11,13].

To improve the editing efficiency of ABE, researchers optimized the codons of ABE7.10, ultimately developing the ABEmax system [19]. In addition, to expand the editing range of ABE, researchers utilized Cas9 protein variants to construct different ABE systems, such as NG-ABE, xCas9-ABE, and ScCas9-ABE. However, these systems showed lower editing efficiency due to their relatively lower compatibility with ABE [32,33].

Subsequently, David R. Liu’s team significantly enhanced the editing efficiency of the base editor by utilizing a phage-assisted evolution screening system to artificially evolve TadA-7.10, ultimately yielding ABE8e. This sophisticated system boasts superior deaminase activity and an expanded editing window. However, the elevated enzyme activity of ABE8e has led to a notable increase in off-target effects. To mitigate this concern, they incorporated the V106W point mutation into TadA, aiming to minimize off-target editing capabilities [23]. In 2022, Tan et al. combined TadA8e with the Cas9 variant Cas9-NG to create PhieABEs, demonstrating high-efficiency editing capabilities [24]. Further research has shown that modifying TadA in ABE can alter the editing characteristics, reducing off-target effects [22]. Notably, in a study conducted by Li et al., the AKBE system, developed using the Cas9 variant nSpRY and TadA8e, demonstrated a significant 41% efficiency in A-to-G editing along with A-to-T editing. Although the majority of A-to-T and A-to-C edits were characterized as indels, the initial generation of T1 exhibited stable inheritance, indicating its reliability and reproducibility [34].

### 3.3. Dual Base Editor

To enhance the efficiency of base editors and enable concurrent modifications of both pyrimidines and purines, researchers have integrated the functions of cytidine deaminase and adenine deaminase onto the nCas9 protein, drawing inspiration from the principles underlying CBE and ABE technologies. This innovative approach has led to the development of a dual base editor, designated as the Adenine and Cytosine Base Editor (ACBE). This editor, comprising UGI, cytidine deaminase, adenine deaminase, and nCas9, is capable of executing both C–G to T–A and A–T to G–C nucleotide conversions. Notably, three distinct versions of ACBE have been reported in the literature: SPACE, A&C-BEmax, and Target-ACEmax (Table 1) [14,25,26]. Rigorous testing of these ACBEs in both plant and mammalian cells has revealed that regardless of the diverse cytidine deaminase and adenine deaminase fusions employed, they exhibit notably low rates of off-target effects [14]. To broaden the application of the dual base editor in plants, researchers fused hAPOBEC3A and ecTadA:ecTadA* together at the N-terminus of nCas9, creating a new dual base editor named STEMEs [14]. Studies have shown that in rice, the STEMEs editor achieves an editing efficiency of up to 15% for simultaneous C–G to T–A and A–T to G–C conversions [14]. In 2021, Wang and his colleagues successfully integrated TadA8e with the N-terminus of nCas9, introducing a novel dual base editor known as pDuBE1. This dual base editor exhibited remarkable efficiency, achieving concurrent editing efficiency of up to 49.7% for C–G to T–A and A–T to G–C transitions in rice, representing a significant advancement in the field of genome editing [27]. Recently, Zhang and his team further enhanced the editing capabilities of ABE, CBE, and ACBE through the design and screening of TadA homologs. They successfully reduced the off-target effects of ACBE and broadened the application scope of base editors, contributing to ongoing advancements in the field [22]. Additionally, Neugebauer et al. developed the dual base editor TadDE, leveraging TadA, which effectively edits cytosine and adenine [30].

### 3.4. Glycosylase Base Editor

Both CBE and ABE have demonstrated widespread utility in the biological sciences, enabling transformations among pyrimidines and purines to advance gene editing. However, the Glycosylase Base Editor (GBE), introduced in 2021, represents a significant advancement by enabling conversions between purines and pyrimidines. GBE comprises nCas9, cytidine deaminase, and Uracil-DNA Glycosylase (UNG), marking a notable leap forward in genetic manipulation (Table 1) [15]. Under the precise guidance of sgRNA, the cytidine deaminase enzyme efficiently converts cytosine into uracil. Subsequently, the UNG precisely excises the uracil. Through meticulous DNA repair processes, this method completes the conversion from cytosine (C) to guanine (G). Studies have demonstrated that the Activation Induced cytidine Deaminase (AID)-nCas9-UNG system effectively converts cytosine to adenine (A) in *Escherichia coli*. Furthermore, modifications to this system, such as the APOBEC-nCas9-UNG system, have achieved cytosine-to-guanine conversion in mammalian cells. Researchers have also constructed the CGBE1 system utilizing the rAPOBEC1 (R33A) mutant, in which UNG is replaced by UGI. This system achieves cytosine-to-guanine conversion while significantly reducing off-target editing. Furthermore, the miniCGBE1 system, devoid of UNG, not only achieves cytosine-to-guanine conversion but also reduces the likelihood of indels [15,28,35]. In 2022, Liang and colleagues successfully integrated the CGBE and ABE technologies and established a novel dual base editing system designated as AGBE. This system is characterized by its remarkable capability to simultaneously facilitate conversions among four distinct types of bases [36]. Subsequently, Tong et al. made significant advancements by linking uracil-DNA glycosylase (UNG) with ABE, culminating in the development of the adenine base editing flipper AYBE. This sophisticated tool exhibits the ability to execute A-to-T and A-to-C conversions within mammalian cells, marking a significant milestone in gene editing [29]. However, the editing efficiency of glycosylase base editor in rice compared to adenine base editors is relatively unstable, greatly limiting their application. Researchers conducted a thorough investigation into the editing efficacy of glycosylase base editors in rice, incorporating a range of TadA8e variants. The study revealed that the editor system, comprising TadA-CDd and OsUNG, demonstrated a superior capacity for C-to-G editing in rice [37].

## 4. Current Status and Methods of Improving Base Editors

Base editing technology represents a significant milestone in genetic editing, allowing precise base substitutions without causing double-strand breaks (DSBs). However, its widespread implementation faces challenges related to the selection of appropriate editing sites, the delineation of editing windows, and the management of off-target effects. To address these challenges and enhance the efficiency and precision of base editing technology, researchers have embarked on improvements and optimizations in various aspects, including the targeting of Cas proteins, the interaction of R-loops with deaminases, and the regulation of editing windows.

### 4.1. Strategies for Enhancing Editing Sites and Windows

The requirement for PAM sequences in base editors limits their broader and more efficient application. In 2017, David R. Liu’s team combined rAPOBEC1 with engineered variants of Cas9, resulting in several base editors that can utilize PAM sequences other than NGG [38]. The following year, they created xCas9, which can recognize NG, GAA, and GAT PAM sequences, and combined it with BE3 and ABE7.10. These combinations could target and edit previously inaccessible regions, with xCas9 (3.7)-ABE showing higher editing efficiency than ABE7.10 [39].

In 2020, Miller and colleagues derived a novel variant of SpCas9 through directed evolution, which is not constrained by PAM sequences. When coupled with a deaminase, this variant can specifically target and modify genetic loci even in the absence of a PAM sequence [14]. Additionally, researchers have developed PAM recognition systems that can target NNG and NAAA sequences, based on ScCas9 and SmacCas9, respectively [33,40,41]. To enhance the efficiency and precision of base editing, modifications to the Cas9 protein are conducted in addition to expanding the number of editing sites. In 2019, David R. Liu’s team aimed to strengthen the interaction between deaminase and single-stranded DNA by fusing various SpCas9 variants with deaminases. This approach led to the development of two CP-CBEmax variants, which exhibited comparable editing activity to CBEmax but with an expanded editing window and superior editing product purity [42]. In the context of the ABE framework, the CP-ABEmax architecture, constructed using similar techniques, demonstrated a substantially enlarged editing window. Both CP-CBEmax and CP-ABEmax exhibited reduced indel rates [43]. In 2024, Yarra and colleagues developed a modified version of SpCas9, designated as SpG, which was subsequently utilized in cytosine base editing systems. This innovation extended the targeting capabilities within the carrot genome and increased the number of editable loci [44]. In cotton, Wang et al. replaced nCas9 with dCpf1, which recognizes TTTV PAM sequences, expanding the targeting range of ABE7.10 and ABE8e [45]. In the same year, Li et al. constructed the AKBE system using the Cas9 variant nSpRY. In rice and tomato, the editing efficiency of A-to-T conversion was 25% and 10.5%, respectively, while achieving A-to-G editing simultaneously, with an average efficiency of 41% [34].

Researchers have also broadened the editing window by altering the interaction between deaminase and DNA. In 2014, they connected multiple GCN4-containing SunTaq tags to the N-terminus of nCas9, creating BE-PLUS [46,47]. In 2019, they fused cytidine deaminase within the PAM domain of the Cas9 protein, resulting in BE-PIGS, which also showed an expanded editing window for base editing [48]. In the subsequent year, scientists integrated adenine deaminase into specific loci of Cas9, yielding several iterations of the ABE-nSpCas9-DS base editor. This not only broadened the editing scope but also exhibited reduced off-target effects [49]. Furthermore, Ryu and colleagues achieved the ABE7.10 base editor through the elongation of the 5′ terminus of the sgRNA, shifting the editing window 2–3 nucleotides away from the PAM sequence [50]. Conversely, Villiger et al. replaced the HNH domain in the Cas9 protein with TadA, resulting in an adenine base editor with an editing window closer to the PAM sequence [51]. In 2024, researchers employ the unique properties of FrCas9, including its 5′-NNTA-3′ PAM density, to develop cytosine and adenine base editors tailored for rice. These editors’ enhanced ability to recognize multiple target sites allowed precise multisite editing in rice [52].

In situations requiring extremely precise target site editing, overly large editing windows can lead to unnecessary base substitutions, reducing the efficiency of precise editing. Therefore, smaller editing windows are needed in such cases. Jeong et al. achieved this by designing the structural domains of base editors, developing various foundational editors to narrow the editing window [53]. Additionally, substituting deaminases can also narrow the editing window of CBEs [54,55,56]. For example, Zhao et al. achieved single-window base editing of ABE and CBE by designing and screening igRNAs to narrow the editing window [57].

### 4.2. Approaches to Optimize Editing Efficiency

Base editors have been widely applied in gene editing, but their off-target effects have always been a pressing issue. In 2015, Kim et al. systematically studied the off-target effects of base editing and developed the Digenome-seq (Digested-genome sequencing) method, which employs high-throughput sequencing to detect off-target sites across the entire genome [58]. Subsequent studies have used similar methods to evaluate the off-target effects of ABE [59,60]. In 2021, Lei et al. further improved off-target detection technology by developing the Detect-seq (dU-detection enabled by C-to-T transition during sequencing) method, which uses chemical labeling combined with biotin pull-down to allow more precise detection of off-target rates of base editors [61]. Base editor editing capabilities are influenced by the performance of various nucleotide-modifying enzymes. In 2024, Yu et al. found that, compared to adenine base editors, both cytosine base editors and glycosylase base editors exhibited relatively unstable editing efficiencies and patterns at different genomic loci in rice, greatly limiting their application [37]. They studied different TadA8e variants for their base editing activity in rice and found that in cytosine base editors, using TadA-CDd and TadA-E27R/N46L led to more efficient C-to-T editing [37]. In contrast, in the glycosylase base editor, the editor system composed of TadA-CDd and OsUNG showed higher C-to-G editing efficiency in rice [37]. If the editing system is built with the TadDE protein, it can achieve efficient editing of both C-to-T and A-to-G [37]. These results indicate that derivatives of TadA8e can enhance the editing efficiency of cytosine base editors and dual base editors in rice. Similarly, to reduce the off-target effects and bystander editing rates of base editors, researchers improved the editing precision of base editors by designing mutations in the deaminase hAPOBEC3A to develop variants of haA3A-CBE. This base editing system achieved up to 58.1% editing efficiency in mouse liver tissue cells and exhibited minimal bystander editing [31].

With the steady advancement of off-target detection methodologies, it has been established that, apart from eliciting off-target modifications, base editors can trigger certain base substitutions as well as indel mutations. Notably, base substitutions are predominantly observed in CBEs, due to the relatively limited activity or narrower range of cytidine deaminase compared to uracil DNA glycosylase [13]. Indel mutations are primarily caused by the removal of the deaminated base, resulting in DNA deletions or insertions, which are more common in CBEs than in ABEs [39]. To reduce the rate of base substitutions and indels, researchers have attempted to increase the quantity of uracil DNA glycosylase inhibitors in CBEs to enhance editing precision [47]. In addition, it has been observed that sometimes base editors not only edit the target base but also affect neighboring bases, a phenomenon known as bystander editing. In 2020, Lee et al. modified the deaminase by altering its preferences, empowering it to edit only the second C in a CC sequence, thereby reducing the likelihood of bystander editing [55]. Following this, Wang et al. further optimized this strategy and developed an A3A version, which showed higher target base editing efficiency and lower bystander editing [62]. Zhao et al. modified the gRNA and designed igRNA to replace the original gRNA. The results showed that using igRNA to guide CBE and ABE significantly improved editing efficiency, achieving near-perfect single-base editing at some loci [57]. In addition, the CBE and ABE systems modified with FrCas9 showed no bystander editing and relatively low indel rates, with 32.5% and 0% indels, respectively, in CBE and ABE [52].

The basic principle of cytosine base editors (CBEs) involves linking the Cas9 protein with cytidine deaminase and UGI, forming the CBE system. In contrast, glycosylase base editors (GBEs) replace UGI with UNG to achieve the conversion of C to G. Chen et al., in their study on mammals, found that both CBEs and GBEs produce relatively high levels of indels, and these indels do not show a clear association with the Cas9 protein [63]. Research has shown that TadA adenine deaminase exhibits higher editing precision compared to cytidine deaminase. Therefore, through screening and evolution, the TadA-8e enzyme, which has the lowest off-target efficiency, was obtained. This enzyme was used to replace the original cytidine deaminase, leading to the construction of the Td-CBE and Td-CGBE systems. These systems demonstrated higher editing efficiency and lower indel rates compared to the original systems [63].

## 5. Application of Base Editors in Crop Genetic Improvement

Base editors have emerged as a gene editing tool of significant practical importance, getting extensive attention in the fields of molecular breeding and species enhancement in plants. Given the successful utilization of base editing technology in microbial systems, researchers are eagerly pursuing its application in plant genetic advancement to enhance plant resilience, nutritional value, productivity, and other desirable traits. Currently, the application of base editors in plants is mainly focused on major crops such as rice, wheat, and maize [64,65].

In 2017, Zong et al. fused Cas9 endonuclease with cytidine deaminase to construct the nCas9-PBE system, achieving up to 43.48% editing efficiency in rice, wheat, and maize [20]. This breakthrough demonstrated the potential of base editing technology in crop improvement. In a study on maize germplasm, Zhong et al. used the optimized AYBE editor, ZmAYBEv3, to target and edit the ZmGA20ox3 gene in maize, resulting in plants with a dwarf phenotype [66]. This application highlights the practical benefits of base editing in modifying specific genes to achieve desired agronomic traits.

The *OsALS* gene in rice encodes acetolactate synthase, a crucial enzyme in plant stress resistance. Studies have shown that specific mutations at the 95th position glycine (G) to alanine (A) or at the 96th position alanine (A) to valine (V), enhance rice tolerance to imidazolinone herbicides [67,68]. The absence of this gene leads to rice plant death. In 2019, Wang et al. used an optimized CBE base editing system to mutate cytosine at position 1882 of the OsALS gene to thymine, obtaining the S627N allele, which confers enhanced herbicide tolerance in rice [10]. In addition, mutation at position 628, changing glycine to tryptophan (G628W), resulted in a new herbicide-resistant allele that further enhances rice tolerance to herbicides [69]. Similar strategies have been employed to regulate *ALS* genes in other plants, such as Arabidopsis, watermelon, and wheat, to enhance herbicide tolerance [65,70,71]. For instance, single-base mutations in the ASL gene have also been reported in soybeans [72]. In this study, Niu et al. used dCas12-engineered BE4max to simultaneously target and edit the GmALS1 and GmALS3 genes in soybeans, successfully obtaining herbicide-resistant mutants *als1/als3*, which harbors a single-point mutation P178S in GmALS1 and P172S in GmALS3 [72].

Additionally, researchers have used base editing technology to modify the OsSLR1 gene in rice, enhancing resistance to lodging [73,74]. Similarly, Xu et al. used base editing to modify the Wx gene, adjusting the amylose content in rice [75,76,77]. In 2024, Yu et al. achieved efficient C-to-T and A-to-G editing in rice using the TadA8e variant-based CBE and GBE systems [37]. Wang et al. expanded the target site range of the ABE8e system by replacing nCas9 with dCpf1. Using this system, they targeted the *GhTFL1* gene in cotton, successfully achieving directed domestication of cotton germplasm. The resulting mutant lines exhibited phenotypes such as moderate height, reduced fruit branch length, and compact plant architecture [45]. In the same year, Zhang et al. successfully used TALE-DdCBE to target-edit the nuclear and chloroplast genomes of rice and tobacco, providing a new approach for using base editors to edit plant organelle and nuclear DNA [78].

In wheat, resistance starch content is directly correlated with amylose content. Starch branching enzyme (SBE) genes are crucial for amylopectin synthesis, and suppressing their expression increases resistant starch levels. Using base editing technology to edit the TaSBEIIa starch branching enzyme gene has resulted in wheat varieties with high resistant starch content. Resistant starch is not easily digested or absorbed, effectively controlling blood sugar and promoting vitamin absorption. Therefore, base editing of TaSBEIIa using base editors can create functional new wheat varieties with enhanced health benefits [79].

## 6. Potential Application of Base Editors in *Populus* Breeding

Base editors have been extensively used in plant genetic editing, primarily focusing on herbaceous species, while relatively little research has been conducted on woody plants. A significant milestone was achieved by the team led by Qiping Qi at the University of Maryland, who successfully established a base editor in poplar trees. This achievement opens new opportunities for base editing in forestry breeding and enhancement efforts [80]. In addition, Yao et al. utilized a Cas9 nickase (nCas9)-based cytosine base editor (CBE) to precisely introduce premature stop codons through C-to-T conversion in the target gene PLATZ, which encodes a transcription factor involved in the response to fungal pathogens in the hybrid poplar clone “717-1B4”. This approach achieved an editing efficiency of 13–14% [81]. In Qi’s group, they employed two cytosine base editors, PmCDA1-BE3 and A3A/Y130F-BE3, as well as an adenine base editor, ABEmax_V1/V2, to carry out base editing at distinct loci within the 4CL1 and PII genes of poplar trees. Their findings revealed that A3A/Y130F-BE3 exhibited a broader editing window and superior editing efficiency in both genes compared to PmCDA1-BE3. Furthermore, they observed comparable editing windows with the adenine base editor within the 4CL1 gene and noted that the use of the AtU3 promoter resulted in higher editing efficiency than the AtU6 promoter [80]. Moreover, their experiments did not reveal any instances of off-target or incidental editing events. This investigation verifies the effectiveness and safety of base editors in *Populus* and provides a powerful tool for studying gene function and genetic trait improvements.

## 7. Discussion

Forestry plays a crucial role in providing economic products, such as timber, paper, and biofuels, while also serving as a carbon sink and aiding in ecological conservation. However, forestry faces multiple challenges, including climate change, pests and diseases, low productivity, and limited genetic diversity. To enhance tree traits, adaptability, and the quality and efficiency of forestry, the development of novel and efficient gene-editing tools is urgently required.

One such promising tool is the base editor, which allows precise modification of target genes at the single-nucleotide level without causing double-strand breaks. By mimicking natural evolution through single-base changes, base editors can improve plant agronomic traits. Since its development, this technology has garnered widespread attention in the field of molecular breeding and species improvement for various crops. Base editors have already been successfully applied in crops like rice, wheat, and maize, enhancing traits such as herbicide resistance, starch content, and lodging resistance.

However, the utilization of base editors in forestry is still limited. The research team led by Yi-Ping Qi has implemented base editors in poplar trees, thus establishing a precedent for their utilization in tree breeding and enhancement. Nevertheless, numerous challenges and opportunities require further exploration and development. These include broadening the scope and precision of base editors to target a wider array of genes and bases, assessing the phenotypic manifestations and biosafety implications of base-edited plants, extending the application of base editors to other economically significant or endangered tree species, integrating base editors with other gene editing technologies or breeding methodologies to generate novel or enhanced traits, and addressing ethical and regulatory considerations pertaining to gene editing in forestry.

Moreover, many crucial genes and significant traits related to the molecular genetic breeding of trees remain to be discovered and harnessed through base editing technology. These include genes involved in lignin biosynthesis, stress tolerance, and growth and development. The development of more efficient and precise base editors, coupled with their application in tree breeding research, will significantly propel the field of tree genetic improvement forward. We believe that base editing technology has the potential to bring transformative advances in the genetic improvement of trees.

## 8. Conclusions

Significant breakthroughs in base editing technology have been made in genetic engineering, with the potential to transform crop and forest breeding. The development of Cytosine Base Editors (CBEs) and Adenine Base Editors (ABEs) has made base editors more efficient and dependable. Their application in key crops like rice, wheat, and maize, as well as in tree breeding, holds great promise for sustainable agriculture and forestry. These advancements pave the way for enhanced plant traits, improved resilience, and increased productivity, contributing to more sustainable and efficient agriculture and forestry.

## Figures and Tables

**Table 1:** Types of base editors and their editing characteristics.

	Base Editor	Deaminase	Cas Protein	Editor Features	References
Cytosine base editor	BE1	APOBEC1	dCas9	C–G to T–A	[11]
	BE3	APOBEC1	nCas9		[11]
	BE4	APOBEC1	nCas9	An additional UGI is connected next to the UGI of the BE3 to improve efficiency	[18,19]
	nCas9-PBE	APOBEC1	nCas9	Editing efficiency up to 43.5%	[20]
	Target-AID	PmCDA1	nCas9		[21]
	miniCBE	TadA8e	nCas9	High accuracy and minimal off-target effects	[22]
Adenine base editor				A–T to G–C	
	ABE7.10	ecTadA	nCas9		[13]
	ABEmax	ecTadA	nCas9		[19]
	ABE8e	TadA8e	nCas9		[23]
	PhieABEs	TadA8e	nCas9-NG	The editing window is extensive	[24]
Dual base editor				Edit K–G–-T to T–A and A–T to George-K at the same time	
	ACBE	PmCDA1 ecTadA:ecTadA	nCas9		[25]
	SPACE	PmCDA1 ecTadA*	nCas9		[26]
	STEMEs	hAPOBEC3A ecTadA:ecTadA*	nCas9	The efficiency of simultaneous editing of C–G to T–A and A–T to G–C is up to more than 15%.	[14]
	pDuBE1	TadA8e	nCas9	In rice, there is a co-editing efficiency of up to 49.7%.	[27]
Glycosylase base editor				Conversion between purines and pyrimidines	
	GBE (AID-nCas9-Ung)	AID	nCas9	Convert C to A	[15]
	GBE (APOBEC-nCas9-Ung)	APOBEC1	nCas9	Convert C to G	[15]
	CGBE1	APOBEC1 (R33A)	nCas9	Low off-target rate	[28]
	AYBE	ABE8e	nCas9	A to T and A to C conversions	[29]
